# Amputation of Leg

**Published:** 1884-04

**Authors:** U. C. Lynde


### Amputation of Leg.

By U. C. Lynde, M. D.

### AMPUTATIONS OF THE LEG IN ITS CONTINUITY.

Of all the amputations made in continuity in the two extremities, that of the leg requires the most care, time and experience. As there are two bones, one small and the other large, care is required while sawing them through that the greater is not cut shorter than the smaller, and that the smaller is sawed through first; and as it is after these amputations that erysipelas, necrosis and pyæmia are most common, therefore, the greatest care is requisite that no spiculæ of bone are left; that the periosteum is uninjured; and, in cases of amputation for injuries, that no soft parts that have been in any way seriously injured are allowed to constitute any portion of the stump. In doing this, sawing off the spine of the tibia, and in securing the vessels here, more time is required than in making amputations elsewhere in continuity in either extremity. Here the surgeon has the sharp spine of the tibia covered anteriorly with nothing but skin, while posteriorly, especially in the calf of the leg, he has a superabundance of soft parts. From this unequal distribution of the soft parts, he must form a covering for a bone, the most irregular in shape, and the most liable to disease, specific and traumatic, of any of the long bones. This stump must bear the greatest amount of pressure of any that the surgeon makes. These facts render experience especially valuable in selecting the site from which the flaps are to be chosen under different circumstances, the number of flaps, and their shape, length, breadth and thickness.

### MORTALITY COMPARED WITH THIGH AMPUTATIONS.

In comparing the mortality of this amputation with that of the thigh, the fact must not be overlooked that while a large percentage of all the fatal cases of the latter can be ascribed to shock, it is very seldom that death, from amputation of the leg, is owing to this cause. Setting aside the cases in thigh amputations that prove fatal in consequence of shock, the mortality is but little more than that of the leg.

### TOURNIQUET USED.

The best way to control hemorrhage while the operation is being made, that I am acquainted with, is by means of the rubber bandage. When ready to operate, I elevate the foot (if it is not too much mangled and its condition does not forbid) for a minute or so, and while it is still elevated, I apply, tightly, the bandage just above the knee. Properly applied, it controls the circulation perfectly, enabling the surgeon to complete the operation without the loss of an ounce of blood.

### METHODS OF FORMING FLAPS, ETC.

The method of amputating, in the leg, varies, with some surgeons, with the particular site at which the amputation is made, some choosing the circular amputation at the lower third, while making the flap operation above, some only making the flap operation, whatever the site. Others confine themselves to the circular operation; while others, still, operate by the modified circular, or the modified flap. Some, in making their flaps, choose them from the sides, others from the anterior and posterior surfaces of the leg. Some make use of but one flap, a long anterior; but the most of surgeons make two flaps; a long posterior and a short anterior; a long anterior and a short posterior; or, a long external and a short internal.

These flaps, too, vary in form and thickness, some only making the oval, others only the rectangular. Some confine themselves to the use of the skin, while others include more or less of the muscular tissue. All claim the superiority of their particular method. That so many different methods of forming the covering of stumps have been and are now practiced with a tolerable degree of success demonstrates, conclusively, that however wedded to one or the other any surgeon may be, he has an opportunity to choose this or that method, as circumstances

26

demand. The surgeon who would not utilize the sound, soft parts that might be left on either side of the leg, and so content himself with making a single flap operation, thereby saving for his patient one to three inches in the length of his limb, certainly is not conversant with the results following this method of amputating; and though he had never heard or known of an operation being made with one flap, he should know, not only that such an operation would offer a good prospect for a useful stump to his patient, but that it was his duty to confine himself, in any particular case, to the only legitimate means at his command. Should the soft parts in the leg, at any particular point, be sound through its entire circumference, and unsound immediately below this point, or, should the limb be cut completely off, as sometimes happens, leaving the soft parts healthy above, no surgeon can fail to see that in order to secure the greatest length to the stump he must content himself with the circular amputation. Ordinarily the single flap requires an unnecessary shortening of the stump, and for this reason, if for no other, should only be resorted to in exceptional cases. But the greatest objection to this method, especially when the flap is made only from skin, is that of its liability to slough. After injuries, the conservative surgeon will sometimes choose one method, and sometimes another, practicing his favorite method only when circumstances do not forbid it.

My own preference has always been for the flap operation. It has always seemed to me that by this method the relation of the parts to each other, skin to fascia, fascia to muscle, blood vessels and nerves to all, was less disturbed in forming the flaps than by any other method; that in dressing up the stump after the formations of its coverings a nicer apposition of cut surfaces was secured; that in consequence of the latter circumstance an earlier union would take place; that the operation was easier of execution, and more rapidly performed, and that it afforded a better covering to the bone, and consequently it secured for the patient a greater degree of comfort in the use of an artificial limb. In making amputations of the leg, whenever circumstances permitted, I have invariably operated by this method, and that, too, regardless of the site. Circumstances have, in some cases, driven me to make the circular, in others, some modification either of the circular or flap operation. In operating after injuries I have always utilized sound tissue in whatever way and manner I found practicable. No one can fail to see that the long cutaneous flap is more liable to slough than one made of the same length and breadth but with a thicker base; and, therefore, it has only been in those cases in which I had but this, alone, that I have been content to trust to such a covering. In operating on the leg, whenever the state of the parts has been such as to admit of it, I have chosen a short anterior, and a long posterior flap. In making these flaps, I first transfix the limb immediately posterior to the tibia and fibula, and so make my long posterior flap first. In fact, I always make my long flap first, from whatever surface chosen, and in whatever manner I may make it. As it is the chief covering of the stump, it seems to me the surgeon should make it while he is unlimited in his material. If it is to be made by transfixion, there are other reasons for making it first. By making this flap first, all liability of cutting the skin and subcutaneous tissues irregularly at the points of ingress and egress is avoided. The surgeon's mind will not be diverted from a close watchfulness over the guidance of the point of his knife about the bones, and from the proper breadth and length of the flap he is making, to the necessity of piercing the skin at a specific point. The limited flap will be left for the surgeon to make with his knife constantly under his eye from angle to angle. Finally, the flaps can be made in this manner with greater ease, confidence, and rapidity. When material enough is at the command of the surgeon he should cut his flaps long enough to come together without any tension, in the least, having to be exerted by the sutures; and there should be a little to spare, in anticipation of some swelling.

Should the stitches at any time become too tight in consequence of swelling of the flaps, and thereby threaten to destroy the capillary circulation in them, they should be cut, after which, if union has not already taken place, the dressings to be hereafter recommended will hold the flaps in position. When making an amputation through the calf of the leg, if the muscular tissue is well developed, it will be necessary to thin the posterior flap. This will be but the work of a few seconds, and is easily done by laying the flap in the left hand, while with the right the knife can be made to pear off from the inner surface such an amount as is thought necessary. The length of the flaps will be regulated by the size of the limb at the point of amputation. I have always used silk ligatures, and as I know I have never thereby lost a patient, and as they have always fulfilled (when I have applied them properly) every indication, I know of no sufficient reason for changing them for some other material with which I am not familiar. After having secured the blood vessels, the nerves should be looked up and cut short, that they may not have their ends become involved in the cicatrix, or be pressed against the rough end of the bone. In amputating near the ankle, it may be necessary to cut from the inner surface of the posterior flap some of the tendinous parts of the gastrocnemius and soleus muscles. Before closing up the flaps I apply water to their cut surfaces as hot as my hand can comfortably bear. This will prevent all oozing of blood from the smaller vessels. Having brought the ends of my ligatures to the angles of the stump, I close it up by taking four, five, or six sutures, depending upon the size of the flaps. Two strips of old muslin, four to six inches wide, and each consisting of four thicknesses, and long enough to go once and a half times round the stump, the one laid under the end of the stump at right angles to the limb, the other laid on top of the first and at right angles to it, and parallel to the limb, and directly under it, both having been saturated with pure tepid water, constitute my dressing; excepting a piece of old linen, also saturated with water, and folded up and placed directly over the junction of the flaps. This last piece is for cleanliness, and can be changed every hour, if necessary, without any inconvenience. There are two folded up cloths under the limb, and one over the junction of the flaps. It is only necessary, now, to bring the end of the one parallel with the limb over the surface of the stump, after which the two ends of the cloth lying at right angles to the limb are brought over and around it, and all further attention can be trusted to the nurse for the next twenty-four hours. He should be directed to lay the ends of the two main cloths off the stump, and after removing the cloth immediately over the edges of the flaps, to place a like one that is clean in its stead as often as every three hours. Under all, leg and dressings, should be placed a rubber blanket for the protection of the bedding. No adhesive plasters, or bandages of any kind, are ever used. Once a day, or every two days, the large cloths should be removed and clean ones substituted. I have practiced this method, in the main, of dressing amputation wounds for fifteen years and I have yet to lose a patient by reason of it, never having lost a patient from sloughing of flaps, erysipelas, septicæmia, or pyæmia, excepting one, and he had been exposed in the cold for thirty-six hours with his leg crushed before I saw him. He was an employe on the Central railroad, was injured at East Buffalo, and remained in a cold car for the time mentioned. He had had a chill before Dr. Briggs and I saw him, and his crushed leg was greatly swollen. He was taken to his home on Seneca street, where we amputated his leg in the upper third. In a few hours this old man became delirous, and died after three days, evidently of septicæmia.

